# Neuron-astrocyte interaction enhance GABAergic synaptic transmission in a manner dependent on key metabolic enzymes

**DOI:** 10.3389/fncel.2015.00120

**Published:** 2015-04-09

**Authors:** Przemysław Kaczor, Dariusz Rakus, Jerzy W. Mozrzymas

**Affiliations:** ^1^Department of Animal Molecular Physiology, Wroclaw UniversityWroclaw, Poland; ^2^Laboratory of Neuroscience, Department of Biophysics, Wroclaw Medical UniversityWroclaw, Poland

**Keywords:** metabolism, GABAergic synapses, miniature inhibitory synaptic currents, astrocytes, glycogen phosphorylase, glutamine synthetase, hippocampal neurons

## Abstract

Gamma aminobutric acid (GABA) is the major inhibitory neurotransmitter in the adult brain and mechanisms of GABAergic inhibition have been intensely investigated in the past decades. Recent studies provided evidence for an important role of astrocytes in shaping GABAergic currents. One of the most obvious, but yet poorly understood, mechanisms of the cross-talk between GABAergic currents and astrocytes is metabolism including neurotransmitter homeostasis. In particular, how modulation of GABAergic currents by astrocytes depends on key enzymes involved in cellular metabolism remains largely unknown. To address this issue, we have considered two simple models of neuronal culture (NC): nominally astrocyte-free NC and neuronal-astrocytic co-cultures (ANCC). Miniature Inhibitory Postsynaptic Currents (mIPSCs) were recorded in control conditions and in the presence of different enzyme blockers. We report that enrichment of NC with astrocytes results in a marked increase in mIPSC frequency. This enhancement of GABAergic activity was accompanied by increased number of GAD65 and vGAT puncta, indicating that at least a part of the frequency enhancement was due to increased number of synaptic contacts. Inhibition of glutamine synthetase (Glns) (with MSO) strongly reduced mIPSC frequency in ANCC but had no effect in NC. Moreover, treatment of ANCC with inhibitor of glycogen phosphorylase (Gys) (BAYU6751) or with selective inhibitor of astrocytic Krebs cycle, fluoroacetate, resulted in a marked reduction of mIPSC frequency in ANCC having no effect in NC. We conclude that GABAergic synaptic transmission strongly depends on neuron-astrocyte interaction in a manner dependent on key metabolic enzymes as well as on the Krebs cycle.

## Introduction

Gamma aminobutric acid (GABA) is the main inhibitory neurotransmitter in the mammalian adult brain and is derived from glutamic acid by glutamate decarboxylase (GAD) which is widely expressed in two isoforms, GAD65 and GAD67, the former in neurons and the latter one both in neurons and astrocytes (Yoon and Lee, 2014). GABAergic inhibition in the phasic and tonic forms has been extensively investigated in the past decades (Farrant and Nusser, 2005) but its interdependence with cellular metabolism remain poorly understood. Glutamate is a GABA precursor and the main excitatory neurotransmitter in the CNS. It is synthesized from glutamine, which can be considered as astrocytic precursor for both neurotransmitters and is supplied directly by astrocytes where it is catalyzed by glutamine synthetase (Glns; Bak et al., 2006). Under physiological and *in vitro* conditions, Glns is localized mainly in astrocytes but in some pathologies it may also occur in neurons (Norenberg and Martinez-Hernandez, 1979; Derouiche and Ohm, 1994; Suárez et al., 1996). Glucose in the brain is delivered from blood vessels to astrocytes where it might be metabolized to glycogen (Cataldo and Broadwell, 1986; Wender et al., 2000; Kong et al., 2002) or transformed to lactate which, in turn, is released and absorbed by neurons providing them with their primary energy substrate (Magistretti and Pellerin, 1999). Glycogen in astrocytes can be cleaved by glycogen phosporylase (Pyg) and metabolized to lactate and/or provide substrates for Krebs cycle to produce ATP and neurotransmitter precursors. In general, astrocytes, besides their well-established functions in metabolism and homeostasis, have been recognized as effective regulators of synaptic transmission. In particular, astrocytes were shown to be strongly involved in synaptogenesis (Diniz et al., 2012). Interestingly, in a recent study, Suzuki et al. (2011) have shown that astrocyte-neuron lactate transport plays a key role in LTP maintenance and memory formation. Thus, metabolic processes turn out to be critical regulators of synaptic plasticity which is a substrate for learning and memory. Whereas plasticity implicated in classic learning paradigms concerns mainly glutamatergic transmission, recent studies clearly demonstrated that GABAergic synapses also show a marked degree of synaptic plasticity (for review: Flores and Méndez, 2014; Petrini and Barberis, 2014; Wenner, 2014) although its mechanisms and, in particular, involvement of astrocytic and neuronal metabolic enzymes remain poorly understood. Several lines of evidence indicate a variety of interactions between astrocytes and GABAergic system. For instance, Ortinski et al. (2010) have shown that astrocytosis may selectively reduce inhibitory GABAergic currents due to diminished expression of Glns. However, Kang et al. (1998) reported that a direct stimulation of astrocytes potentiated miniature Inhibitory Postsynaptic Currents (mIPSCs) in pyramidal neurons. Interestingly, release of GABA from astrocytes has been implicated in regulation of tonic form of inhibition (Lee et al., 2010). Recently, Christian et al. (2013) have demonstrated that endozepines released from astrocytes potentiate GABAergic currents in RT thalamic nucleus. It is noteworthy that astrocytes express GABA_A_ and GABA_B_ receptors (Lee et al., 2011) indicating that interaction between astrocytes and GABAergic cells maybe reciprocal.

Thus, cross-talk between GABAergic synapses and astrocytes may involve a wealth of potential mechanisms including metabolism control, modulation of balance between neurotransmitters and their precursors and modulation of both neurons and astrocytes by several gliotransmitters (for review by Sahlender et al., 2014). It is thus difficult to precisely indicate the role of specific regulators in the neuronal-astrocytic cross-talk. In particular, how the regulation of GABAergic transmission by astrocytes depends on major enzymes regulating astrocytic metabolism (e.g., Pyg) or neurotransmitter homeostasis (e.g., Glns) remains poorly understood. To address these issues we have considered simple models of neuronal culture (NC): nominally without astrocytes (NC) and neuronal-astrocytic co-cultures (ANCC) and measured mIPSCs in control conditions and in the presence of pharmacological agents blocking enzymes of interest. We report that enrichment of NC with astrocytes results in a marked increase of mIPSC frequency. This increased synaptic activity was accompanied by increase of GAD65 and vGAT puncta co-localizing with cells stained with neuronal marker tubulin β3. Inhibition of Glns (with l-methionine-sulfoxide, MSO) strongly reduced mIPSC frequency in astrocyte-neuron co-culture whereas Glns inhibitor in NC was ineffective. Moreover, treatment of ANCC with inhibitor of Pyg or with selective inhibitor of astrocytic Krebs cycle fluoroacetate (FA), resulted in reduction of mIPSC frequency in ANCC having no effect in NC. We conclude that GABAergic synaptic transmission strongly depends on neuron-astrocyte interaction in a manner dependent on key metabolic enzymes (Glns, Pyg) as well as on the Krebs cycle.

## Materials and Methods

### Cell Culture

All performed procedures on rats were approved by The Scientific Research Ethical Committee (permission#118/2010). Cell cultures were prepared from P0-P1 Wistar rat pups using methodology described in detail elsewhere (Mozrzymas et al., 2011) with slight modifications. Briefly, hippocampi were quickly removed from brains in ice cold dissociation medium DM (in mM: 81.8 Na_2_SO_4_, 30 K_2_SO_4_, 5.8 MgCl_2_, 0.25 CaCl_2_; 1 HEPES, 20 Glucose; 1 Kynureic Acid, 0.001% Phenol Red). Dissected hippocampi were treated twice for 15 min with 100 U papain, rinsed three times in DM and plating medium (MEM, 10% FBS, antibiotics). Next, hippocampal tissue was mechanically dissociated with fire polished glass pipettes. Cell suspension was then transferred to 10 ml OPTI-MEM and centrifuged at 163 g. Obtained cell pellet was then re-suspended in the plating medium and plated at density 3.15 × 10^4^ cells at 18 mm diameter cover slips, covered with poly-Lysine (Sigma-Aldrich, Germany) and laminin (Roche, France). After 2 h, medium was changed to NA/B-27 medium (culture growth medium) (Neurobasal-A w/o Phenol Red, 2% B-27 Supplement, 1% Penicillin/Streptomycin, 0.5 mM Glutamine, 12.5 μM Glutamate, 25 μM β-mercapto-ethanol. After 48 h, to prevent astrocytic growth, AraC was added at a final concentration of 25 μM. Primary cell culture obtained using this protocol contained a small (10–15%) astrocyte contamination. To prepare the astrocyte-neuron co-cultures (ANCC), astrocytes were added to NC at final density of approximately 4 × 10^4^ cells/cm^2^. All electrophysiology and immunofluorescence experiments were performed within 13 and 16 days in culture in the case of ANCC and not earlier than 24 after astrocytes addition to NC (to avoid suppression of neurons by astrocytic overgrowth). Astrocytes were prepared from the same animals as neurons. All cells, after dissociation of hippocampi, were cultured in plastic 25 ml flask in DMEM (IITD, Poland) supplemented with 10% of fetal bovine serum (Gibco, USA), 2 mM glutamine, 5.55 mM glucose (Chempur, Poland) and 1% Penicillin/Streptomycin. To remove remaining fibroblasts, astrocyte growth medium was supplemented with d-Valine and, to eliminate neuronal contamination from astrocyte culture, two passages were performed, once a week before the use in the co-cultures. Unless otherwise stated, all chemicals were from Sigma-Aldrich, Germany.

### Electrophysiological Recordings

GABAergic mIPSC were recorded in the whole-cell configuration of the patch-clamp technique at the membrane voltage of −70 mV, using the Axopatch 200B amplifier (Molecular Devices Corporation, USA). Signals were acquired at 50 kHz following low-pass filtering at 5 kHz using Digidata 1440A (Molecular Devices Corporation) interface and pClamp10 software (Molecular Devices Corporation). The intrapipette solution contained (in mM): 137 CsCl, 1 CaCl_2_, 2 MgCl_2_, 11 BAPTA (tetra cesium salt), 2 ATP and 10 HEPES, pH 7.2 with CsOH. The composition of the external solution was the following (in mM): 137 NaCl, 5 KCl, 2 CaCl_2_, 1 MgCl_2_, 24 glucose and 10 HEPES, pH 7.2 with NaOH. mIPSCs were recorded in the presence of 1 μM tetrodotoxin (TTX) to suppress neuronal excitability and 10 μM Kynurenic acid was added to the external saline to block the glutamatergic currents (in our conditions mainly those mediated by AMPA receptors). The rise time kinetics of mIPSCs was described as 10–90% onset time and the decay time course was fitted with a sum of two exponential functions:

(1) $$T_{dec}\left(t\right)=A_{1}e^{\frac{\tau_{1}}{t}}+A_{2}e^{\frac{\tau_{2}}{t}}$$

where τ_1_, τ_2_ are the time constants and A_1_, A_2_ are respective amplitudes. Based on this fit, the weighted decay time constant was calculated: τ_mean_ = *a*_1_τ_1_ + *a*_2_τ_2_, where *a*_1_, *a*_2_ are normalized amplitudes (*a*_1_ = *A*_1_/(*A*_1_ + *A*_2_), *a*_2_ = *A*_2_/(*A*_1_ + *A*_2_). Detection and analysis of mIPSC time course was performed with Clampfit 10 software (Molecular Devices Corporation, USA). To construct the cumulative distribution, data are first sorted in the ascending order yielding the array of the *X* values. For any *X*, *Y* is determined as the ratio of counts of *X* values which are smaller or equal to the considered *X* and the total number of counts for *X* values. Statistical significance was assessed using unpaired two-tailed *T*-test accompanied by Fisher test. Differences between data sets were considered statistically significant when *p* ≤ 0.05. To test the impact of glycogen phosphorylase (Gys), glutamine synthase (Glns) and aconitase, for each considered enzyme, recordings were performed on cells divided into two groups: control (NCC or ANCC) and cells treated by medium supplemented with respective inhibitors (5 μM BAY U6751 for 30 min; 1 mM-methionine sulfoxide, MSO for 30 min; 1 μM FA for 30 min) and α-Cyano-4-hydroxycinnamic acid (4-cin, a blocker of monocarboxylate transporters, 100 μm for 15 min, to block lactate transport between astrocytes and neurons). After treatment, coverslips with cells were transferred to external solution containing respective inhibitor (at the same concentration as during treatment) to perform the whole cell recordings. All chemicals, unless otherwise stated, were from Sigma-Aldrich-Germany.

### Immunofluorescence

Immunofluorescence staining was performed for NC, ANCC and astrocyte cultures after fixation with 4% PFA (Sigma-Aldrich, Germany) in PBS. Prior to application of the primary antibodies, to prevent non-specific reaction, fixed cells were incubated with 5% BSA in PBS for 30 min. Reactions with primary antibodies were run overnight at 4°C. Secondary antibodies in 5% BSA were applied next day for 1 h. Cell nuclei were visualized with DAPI. After each step, fixed cells were washed with 0.1% triton (BDH Chemicals, England) and three times with PBS. Cultures snapshots were gathered with Olympus fluo-view 1000 confocal microscope (final magnification 600× with objective 60× and ocular 10×) and analyzed with Olympus FV 1000 viewer software and a freeware ImageJ software together with SynaptcountJ, NeuronJ and Bioformats plugins. Changes in the density of synaptic puncta stained with vGAT, gephyrin or GAD65 were assessed by counting their number per single cell stained with neuronal marker tubulin β3, and calculated as puncta/cell. More precisely, the number of puncta was determined only for the cell area clearly stained against tubulin β3. To assess the number of immunostained points we used the highest possible power (600×, objective 60×) but at this magnification only a few neurons could be observed in a single snapshot. To make our assessment of puncta/cell more representative, for each cell culture preparation, 8–10 different snapshots were acquired both for NC and ANCC and for each snapshot the average number of puncta/neuron was calculated. Considering that our data have been collected from five preparations, the NC and ANCC groups were represented by 40–50 values determined from the snapshots. To compare these data sets, the unpaired, two tail *T*-test was applied. Primary antibodies and their dilution: Gephyrin monoclonal mouse 1:50 (Cat. No. 147 111), GAD65 polyclonal rabbit 1:1000 (Cat. No. 198 102), β3-tubulin polyclonal rabbit 1:2000 (Cat. No. 302 302), vGAT polyclonal rabbit crude antiserum 1:1000 (Cat. No. 131 002) (all from Synaptic Systems, Germany), β3-tubulin monoclonal mouse (T5076) 1:1000, GFAP polyclonal rabbit (G9269) 1:1000 and GFAP monoclonal (G6171) mouse 1:000 (all three from Sigma, Germany). Primary antibodies were visualized with FITC 1:1000 (F6005, F5636) or TRITC 1:1000 (T6778, T6528) conjugated secondary antibody against rabbit or mouse (Sigma, Germany).

## Results

### Astrocytes Increase GABAergic Activity in Cultured Hippocampal Neurons

**Figure 1:**
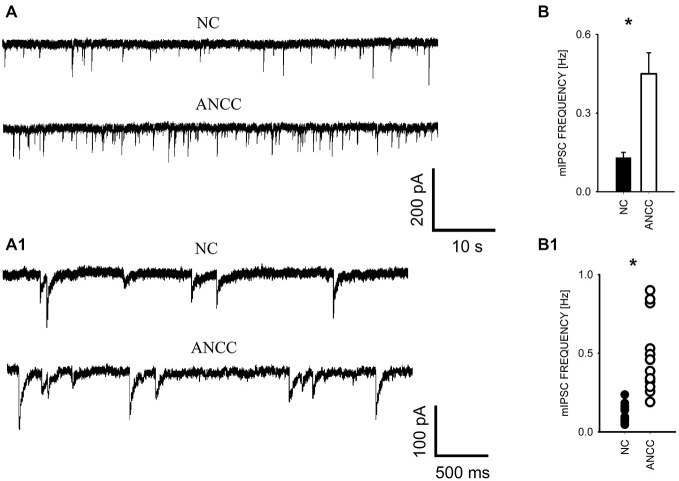
**Supplementation of neuronal culture (NC) with astrocytes strongly up regulates mIPSC frequency. (A)**, Typical traces recorded from neurons in NC and from ANCC. Lower traces in **(A)** show examples of the same recordings in an expanded time scale to show kinetic features of mIPSCs (rapid onset and relatively slow decaying phase). **(B)**, statistics for mIPSC frequency in NC (filled bar) and in ANCC (open bar). **(B1)**, scattered plot of data for NC (filled circles) and for NC (open circles). Asterisks indicate significant difference between respective groups.

**Figure 2:**
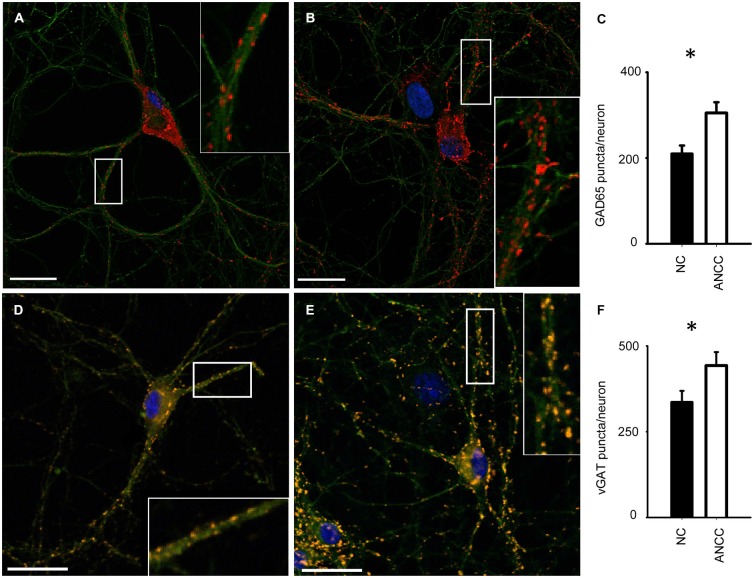
**Supplementation of neuronal culture (NC) with astrocytes increases the number of synaptic puncta. (A,B)**, typical examples of staining for synaptic puncta with antibodies against GAD65 (red) in NC **(A)** and ANCC **(B)**. Green color reflects tubulin β3 staining in all graphs. **(C)** Statistics for the number of GAD65 puncta per neuron in NC (filled bar) and in ANCC (open bar). **(D,E)**, examples of staining for synaptic puncta with antibodies against vGAT (orange) in NC **(D)** and ANCC **(E). (F)**, Statistics for the number of vGATpuncta per neuron in NC (filled bar) and in ANCC (open bar). Asterisks indicate significant difference between respective groups, spacebar in pictures represents 100 μm.

mIPSCs were measured in the whole-cell mode at −70 mV and in the neuronal cell culture (NC) the averaged current frequency was 0.13 ± 0.02 Hz (*n* = 15) and the mean mIPSC amplitude was 50.72 ± 2.43 pA (*n* = 15). Time course was characterized by a rapid onset (10–90% rise time = 1.51 ± 0.05 ms, *n* = 15) and a biphasic decay with the weighted decay time constant (39.89 ± 1.4 ms, *n* = 15). Interestingly, addition of astrocytes to the NC resulted in a dramatic (nearly fourfold, Figures 1A/A1) increase in mIPSC frequency to 0.42 ± 0.079 Hz (*n* = 15) in ANCC (*p* < 0.05) when recordings were made at least 24 h after addition of astrocytes (See Section Materials and Methods). However, addition of astrocytes did not affect the mean mIPSC amplitude (49.39 ± 4.24 pA, *n* = 15, *p* > 0.05). Moreover, the time course of mIPSCs (10–90% rise time and weighted decaying time constants) were not significantly altered in the ANCC with respect to those determined for NC (data not shown, *p* > 0.05, *n* = 15). The results of electrophysiological analysis suggest that the presence of astrocytes does not affect the postsynaptic properties of mIPSCs (amplitude and time course) and therefore the increased frequency might result either from increased number of GABAergic synapses and/or increased quantal release. To address the former possibility, we have performed the immunofluorescence on five different cell lines, staining for GAD65 (glutamate decarboxylase). This staining revealed a significant 45% increase in GAD65 puncta in ANCC neurons (210 ± 19 puncta/cell, *n* = 44 in NC and 305 ± 25 puncta/cell, *n* = 50 in ANCC, *p* < 0.05). To provide further evidence that astrocyte enrichment gives rise to increased number of synapses, immunofluorescence staining was additionally made for vGAT (GABAergic presynaptic marker) and gephyrin (key postsynaptic scaffold protein of GABAergic synapse). Analysis of vGATpuncta confirmed their significantly increased number in ANCC by 31% Figure 2 (336 ± 33 puncta/cell, *n* = 43 in NC and 443 ± 39 puncta/cell,* n* = 46 in ANCC, *p* < 0.05). Increase in number of gephyrin spots in ANCC compared to NC, was slightly above the border line of statistical significance level (*p* = 0.073 data not shown). These data indicate that enrichment of the NC with astrocytes results in an enhanced mIPSC frequency which is accompanied, to a smaller extent, by increased number of GABAergic synaptic puncta.

### The Effect of Glycogen Phosphorylase Inhibition

**Figure 3:**
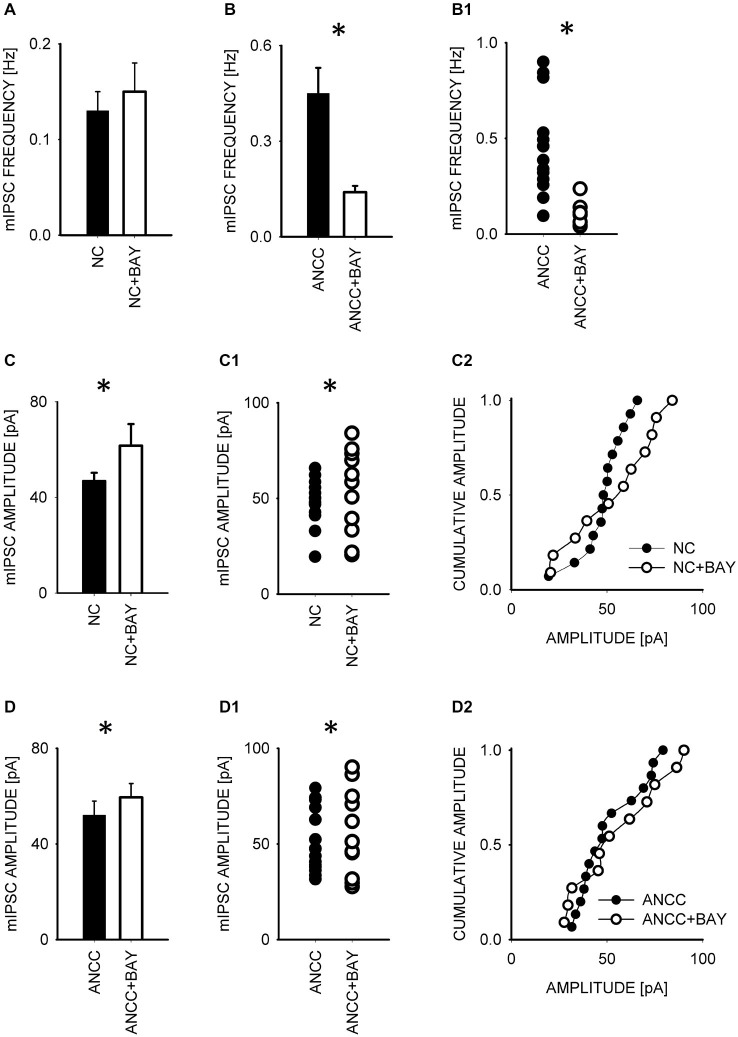
**Blocker of glycogenphosphorylase, BAY reduces mIPSC frequency in ANCC and induces a slight increase in mIPSC amplitude both in NC and ANCC**. Frequency of mIPSCs in NC is not affected by BAY treatment **(A)** but in ANCC a strong decrease in mIPSC frequency is observed in the presence of this compound **(B)**. In **(B1)**, scatter plot of data for which averaged values are shown in **(B). (C)**, statistics for BAY effect on mIPSC amplitudes in NC. **(C1,C2)** show scatter plot and cumulative distribution, respectively for data for which averages are presented in **(C). (D)**, statistics for BAY effect on mIPSC amplitudes in ANCC. **(D1,D2)** show scatter plot and cumulative distribution, respectively for data for which averages are presented in **(D)**. Asterisks indicate significant difference between respective groups.

**Figure 4:**
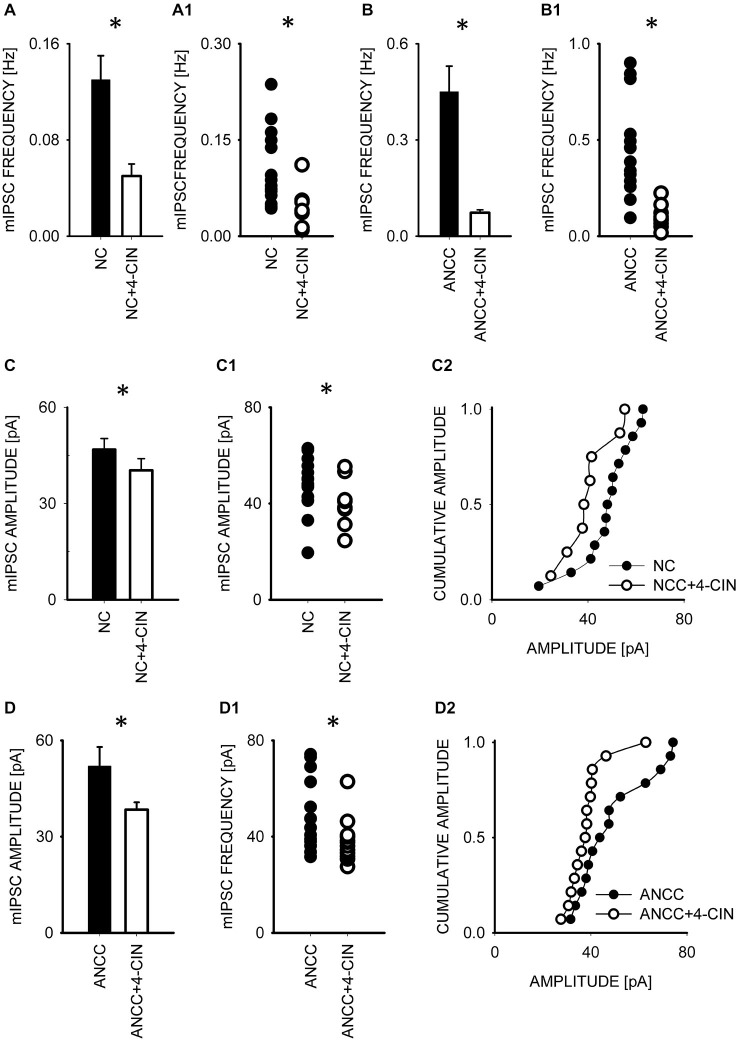
**Blocker of MCT transporters, 4-cin, reduces mIPSC frequency and amplitudes both in NC and ANCC. (A,B)** Statistics of 4-cin effect on mIPSC frequency in NC **(A)** and in ANCC **(B)**. In **(A1,B1)**, scatter plots are presented for data for which averaged values are shown in **(A,B)**, respectively. **(C)**, statistics for the effect of 4-cin on mIPSC amplitude in NC. **(C1,C2)** show scatter plot and cumulative distribution, respectively for data for which averages are presented in **(C). (D)**, statistics for 4-cin effect on mIPSC amplitudes in ANCC. **(D1,D2)** show scatter plot and cumulative distribution, respectively for data for which averages are presented in **(D)**. Asterisks indicate significant difference between respective groups.

To test whether observed effect of astrocytes on GABAergic currents depended on astrocytic metabolism we first checked the impact of Pyg inhibition on GABAergic synaptic currents. To this end, mIPSCs were recorded in NC and ANCC in control conditions and after incubation with the inhibitor of this enzyme BAY U6751 (5 μM, see Section Materials and Methods). In NC, no effect of BAY U6751 was observed on mIPSC frequency (Figure 3A) or time course (*p* > 0.05, *n* = 10, data not shown). However, this inhibitor strongly reduced mIPSC frequency in ANCC Figures 3B,B1 (0.45 ± 0.008 Hz, *n* = 10 in control; 0.14 ± 0.02 Hz, *n* = 10 in BAY U6751) but, again, did not affect the time course of mIPSC (data not shown). The scatter plot (Figure 3B1) shows that the presence of BAY U6751 has limited the frequency distribution to lowest values observed in ANCC. Moreover, administration of Pyg inhibitor to ANCC caused a reduction of mIPSC frequency which was comparable to the difference betweenmIPSC frequency in ANCC and NC in control conditions. Interestingly, mIPSC amplitude in NC and ANCC was significantly increased following treatment with BAY U6751 (Figures 3C–D2, from 50.77 ± 2.42 pA in control to 67.72 ± 4.44 in BAY U6751 *n* = 10 for NC and from 51.95 ± 4.24 pA in control to 59.5 ± 4.8 pA in BAY U6751 *n* = 10 for ANCC). However, as shown in the scatter plots and cumulative distributions (Figures 3C–D2), the measured values of amplitudes largely overlapped with only a few extra-large amplitude values in the presence of BAY U6751. Lactate, released from astrocytes due to glycogen breakdown, is believed to be taken up by neurons by means of MCT transporters. To verify to what extent observed neuronal-astrocyte cross talk relies on the MCT activity in our model, we used a MCT blocker 4-cin (α-Cyano-4-hydroxycinnamic acid). Application of 4-cin during incubation and its presence during electrophysiological measurements at a final concentration of 0.1 mM resulted in a reduction of mIPSC frequency both in NC and NACC (from 0.14 ± 0.02 Hz, *n* = 15 in control to 0.05 ± 0.01 Hz, *n* = 8 after 4-cin treatment NC and from 0.45 ± 0.008 Hz, *n* = 10–0.1 ± 0.01 Hz, *n* = 12 in ANCC in all cases *p* < 0.05, Figure 4). This reduction was associated with the disappearance of high and appearance of extra low frequency values in the scatter plots (Figures 4A1,B1). Interestingly, treatment with 4-cin significantly decreased also mIPSC amplitudes in both models, however, the 4-cin effect on amplitude was much greater in ANCC than in NC (from 46.88 ± 3.38 pA, to 40.36 ± 3.62 pA, *n* = 8 in NC and from 51.95 ± 4.24 pA to 38.41 ± 2.27 pA in ANCC, *p* < 0.05, Figure 3) but the amplitude ranges in the scatter plots largely overlapped in the two models (Figures 3C–D2). There was no effect of 4-cin on the mIPSC time course both in NC and ANCC (data not shown). Thus, blockade of Pyg affected mIPSCs frequency in ANCC only whereas MCT inhibition reduced mIPSCs frequency both in NC and in ANCC and this effect was accompanied by amplitude reduction in both models.

### The Effect of Glutamine Synthetase Inhibition

**Figure 5:**
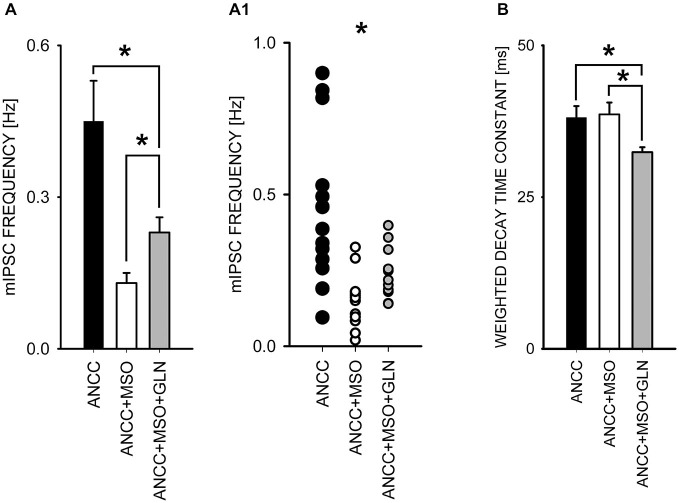
**Block of glutamine synthetase with MSO reduces mIPSC frequency in ANCC. (A)**, statistics of MSO effect on mIPSC frequency (black bar—control conditions, open bar—in the presence of MSO, gray bar in the presence of MSO and 1 mM glutamine). **(A1)** shows a scatter plot for data for which averages are presented in **(A). (B)**, statistics for the weighted decay time constants in the three groups considered in A. Note that in the presence of MSO and 1 mM glutamine, mIPSCs decay is slightly but significantly accelerated. Asterisks indicate significant difference between respective groups.

To check for the impact of Glns inhibition we used l-methionine sulfoxide (MSO) at a final concentration of 1 mM. Treatment with MSO resulted in a significant reduction of mIPSC frequency in ANCC (control—0.44 ± 0.02 Hz, *n* = 15; MSO 0.14 ± 0.02 Hz, *n* = 13, *p* < 0.05, Figures 5A,A1) whereas in NC no significant MSO effect was observed (*p* > 0.05, data not shown). Notably, reduction of mIPSC frequency by MSO in ANCC was similar to that observed between NC and ANCC in control conditions. Treatment with MSO had no effect on mIPSC amplitudes or their time course (data not shown). As already mentioned, Glns mediates catalytic conversion of glutamate to glutamine which is released from astrocytes and taken up by neurons where it is subsequently converted into respective neurotransmitters. Thus, considering this scenario, it is expected that exogenous administration of glutamine should prevent the reduction of mIPSC frequency in the presence of MSO. However, supplementation of the external solution with 1 mM glutamine (concentration in astrocyte growth media) resulted in only a partial, although statistically significant, rescue of mIPSC frequency (to 0.23 ± 0.03 Hz, *n* = 20, *p* < 0.05 Figure 5A). Notably, as shown in scatter plot (Figure 5A1) administration of MSO to NACC resulted in disappearance of high and appearance of extra low frequencies, whereas exogenous glutamine shifted the range of frequencies upward but highest frequency values observed in control ANCC were not present in this group. In addition, although MSO had no effect on mIPSC amplitudes (51.05 ± 6.00 pA, *n* = 15 in ANCC, 47.91 ± 4.72 pA, *n* = 15 in ANCC with MSO and 51.49 ± 2.52 pA, *p* > 0.05, *n* = 20 with glutamine supplementation, *p* > 0.05)or kinetics (data not shown), in the case of MSO treated ANCC, supplementation with 1 mM glutamine resulted in a significant shortening of the weighted decay time constant (for ANCC 38.08 ± 1.93 ms *n* = 15, for ANCC treated with MSO, 38.00 ± 2.92 ms, *n* = 15 and for ANCC treated with MSO and 1 mM glutamine supplementation, 32.4 ± 0.83 ms, *n* = 20; Figure 5B). Taken altogether, Glns inhibition resulted in a reduction of mIPSCs frequency only in ANCC and this effect could be partially reversed by exogenous glutamine administration.

### The Effect of Inhibition Astrocytic Krebs Cycle with Fluoroacetate

**Figure 6:**
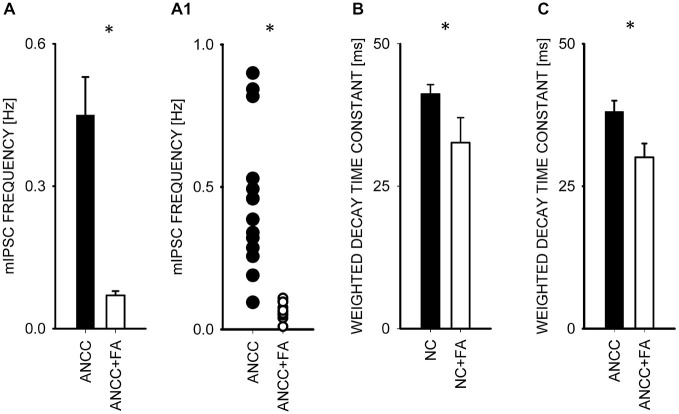
**Block of the Krebs cycle with fluoroacetatereduces mIPSC frequency and accelerates decay kinetics in ANCC. (A)**, statistics of FA effect on mIPSC frequency (black bar—control conditions, open bar—in the presence of FA). **(A1)** shows a scatter plot for data for which averages are presented in **(A). (C,B)** statistics for the weighted decay time constants for controls recorded from ANCC (filled circles) and in the presence of FA (open Bar). Asterisks indicate significant difference between respective groups.

To investigate the impact of Krebs cycle in astrocytes on GABAergic transmission we used fluoracaetate (FA) which is a selective inhibitor of aconitase. Incubation of NC and ANCC in the presence of 1 μM FA strongly reduced the mean mIPSC frequency only in ANCC without any impact on mIPSC frequency in NC (from 0.44 ± 0.08 Hz, *n* = 10 in control ANCC to 0.04 ± 0.005 Hz, *n* = 9, after FA treatment, *p* < 0.05, Figures 6A/A1), without any significant effect on mean mIPSC amplitude in both NC and ANCC and its cumulative distribution didn’t show any significant difference in both cases (data not shown). FA had no effect on rise time of mIPSC in NC or ANCC (data not shown) but it had a statistically significant effect on decay time in both NC (from 41.17 ± 1.65 ms *n* = 10 in control to 32.64 ± 4.38 ms, *n* = 9 in FA, *p* < 0.05, Figure 5B) and ANCC (from 38.25 ± 1.93 ms, *n* = 10 in control to 30.06 ± 2.43 ms, *n* = 9 in FA *p* < 0.05 Figure 6C). Thus, blocking the Krebs cycle by aconitase inhibition resulted in a reduction of mIPSC frequency in ANCC only and, additionally, an acceleration of synaptic current decay both in NC and ANCC was observed.

## Discussion

The most important finding of this study is that astrocytes strongly up regulate GABAergic transmission in the model of cultured hippocampal neurons. This effect was manifested by a nearly fourfold increase in mIPSC frequency while neither the amplitude nor current time course were affected, suggesting a presynaptic mechanism. However, up regulation of mIPSC frequency may potentially involve formation of new synapses and increase in spontaneous release probability of a single synaptic vesicle. Our immunofluorescence staining showed increased number of GAD65 and vGAT puncta in ANCC, providing evidence that the presence of astrocytes up regulates the number of GABAergic synapses. However, it should be noted that increase in synaptic puncta in ANCC was considerably smaller than that for mIPSC frequency. Although immunofluorescence staining cannot be regarded as a strictly quantitative approach (an unknown proportion of synaptic puncta is not detected), it is tempting to propose that it provides a reasonable estimate of increased proportion of synapses in ANCC. This suggests that increased number of synapses might be responsible only for a part of increased mIPSC frequency in ANCC. The mechanism underlying such a strong impact of astrocytes on mIPSCs remains unknown and, in general, astrocyte-neuronal cross talk in the context of mIPSC frequency regulation remains poorly understood. It has been proposed, for instance, that it might be related to the modulation of calcium fluxes affecting the intracellular calcium and thereby the release probability (Araque et al., 1998). It is also known that GABAergic synaptic transmission may depend on glutamate uptake (Mathews and Diamond, 2003; Potapenko et al., 2013) which is strongly supported by astrocytes but in these studies, the major observed effect was on IPSC amplitudes.

Importantly, a strong increase in mIPSCs frequency in ANCC was largely abolished by inhibition of Pyg, Glns or aconitase, underscoring the importance of these key enzymes in neuronal-astrocytic cross-talk. Pyg can be expressed both in astrocytes and in neurons (Pellegri et al., 1996; Pfeiffer-Guglielmi et al., 2003) raising a possibility that inhibition of glycogen cleavage in astrocytes was not the only mechanism underlying the observed effect of BAY U6751 in ANCC. However, BAY U6751 was ineffective in NC indicating that neuronal Pyg was not essential for the observed effect.Interestingly, inhibition of Pyg has also been reported by our group to affect glutamatergic synaptic currents although in that case, blockade of this enzyme reduced mEPSC amplitude with no effect on frequency or time course (Mozrzymas et al., 2011) indicating a different but, as yet, unknown mechanism. It seems plausible, however, that it might involve so called lactate shuttle (Magistretti and Pellerin, 1999) which postulates that glycogen is broken by Pyg to glucoso-6-phosphate and then transformed to lactate, released and taken up by neurons by monocarboxylate (MCT) transporters (Halestrap and Meredith, 2004; Pierre and Pellerin, 2005). It is possible that astrocytes could affect the expression of neuronal MCT in our model although studies performed on layered cultures by Debernardi et al. (2003) did not indicate any clear effect of astrocytes on MCT expression and distribution (Pellerin et al., 2005).

Similar to BAY U6751, 4-cin, a blocker of monocarboxylate transporters, reduced mIPSC frequency in NACC but in contrast to the Pyg inhibitor, it also affected current amplitudes both in NC and ANCC. In another model (retina) it has been reported by Bui et al. (2004) that 4-cin treatment resulted in a decreased GABA, glutamate and glutamine level. It is thus likely that observed here decrease in mIPSC amplitudes might result from reduction of neurotransmitter pool after inhibition of lactate import by neurons. As already mentioned, astrocyte-derived lactate is the major energy source for neurons and it is likely that inhibition of its uptake by neurons triggers redirection of glutamine from neurotransmitters synthesis to mitochondria for energy production and hence, it results in lower GABA content in vesicles. However, mIPSC amplitude reduction was also observed in NC (although to a much smaller extent), where the mechanism based on astrocyte derived lactate is not expected to occur. Nevertheless, as mentioned in Methods, our primary neuronal cell cultures could contain a small (10%–15%) astrocytic contamination which might potentially support a residual of effect observed in NACC.

Interestingly, BAY treatment caused an increase of mean mIPSC amplitude both in ANCC and in NC (Figure 3). Sickmann et al. (2012) have reported that the block of glycogen degradation in diabetic rats resulted in an increase of GABA pool, a result compatible with our observation of increased mIPSC amplitude. It should be also considered that in contrast to 4-cin, which regulates the entrance of lactate into neurons, BAY U6751 affects the intracellular production of glycogen-derived intermediates, (i.e., not only lactate) and, it is not surprising that their effects could be different. Although astrocytes are regarded as a main source of glycogen-derived metabolites in NACC, it was also shown that neurons may synthesize and degrade lactate to a modest extent (Suzuki et al., 2011; Hertz et al., 2014). Thus, it might be hypothesized that glycogen-derived lactate, both from astrocytes and/or neurons, is somehow involved in the regulation of GABA/glutamate ratio in neurons. However, there are studies showing that lactate does not play any substantial role in the regulation of neuronal excitability (Ivanov et al., 2014). It cannot be excluded that lactate and enzymes involved in the Krebs cycle might affect the reversal potential for GABA-induced currents (e.g., Holmgren et al., 2010; Tyzio et al., 2011) and thereby to affect the network activity. This issue would require a thorough measurements using perforated patch technique. We plan to pursue this issue by using the perforated patch recording along with the ultrastructural studies and quantal analysis.

In our experiments the blockade of Glns with MSO reduced mIPSC frequency in NACC but not in NC and theiramplitudes in both models were unchanged. The lack of MSO effect on mIPSC amplitudes indicates that vesicular GABA content was not affected. This observation suggests that neurons are endowed with efficient buffering systems, capable to assure physiological quantal size, even in conditions of prolonged glutamine deficit. Presumably the inhibition of glutamine turnover between astrocytes and neurons did not affect, within the time scale of our experiments (1 h), GABA content in synaptic vesicles, but the deficit of astrocyte-derived glutamine reduced the pool of readily releasable vesicles and decreased the number of functional synapses. Precise estimation of contributions from these mechanisms will require further investigations focused on synaptic mechanisms. Interestingly, supplementation of MSO containing medium with glutamine rescued the mIPSC frequency only partially (Figure 5A), although glutamine concentration used in these experiments (1 mM) was markedly larger than that measured in hippocampal area (193.4 μM; Lerma et al., 1986) and cortical region (385 ± 16 μM; Kanamori and Ross, 2004). However, Boulland et al. (2002) have reported that GABAergic, glutamatergic and glycinergic synaptic boutons are ensheated by astrocytic processes characterized by a particularly high expression of glutamine transporter SN1. Thus, it is possible that exogenous glutamine administration does not fully mimic specific conditions characterizing subtle interactions between tiny astrocytic processes and the presynaptic boutons, where glutamine transfer is expected to be crucial. Another of our observations indicating that supplementation with exogenous glutamine does not fully reproduce astrocyte-neuronal exchange of this aminoacid is the fact that glutamine administration in the presence of MSO affected mIPSC decay kinetics both in NC and in NACC (Figure 5B) whereas these parameters in control conditions and upon MSO treatment were indistinguishable. Thus, administration of glutamine seems to trigger an additional modulatory effect resulting in shorter mIPSC duration. The mechanism underlying this observation remains unknown.

In our study we found that FA, a compound blocking Krebs cycle enzyme, aconitase, and thereby ATP synthesis, strongly down regulated mIPSCs frequency. Importantly, FA is taken up mainly by astrocytes (Muir et al., 1986; Paulsen et al., 1987; Hassel et al., 1992). Just recently, it has been demonstrated that ATP released from astrocytes may increase mIPSC by activation of metabotropic purynergic receptors (Torres et al., 2012). Thus, our findings of a strong FA-induced depression of GABAergic activity in ANCC could reflect the abolition of ATP release from astrocytes. Also it is worth mentioning that FA can affect glutamine-glutamate cycle by preventing glutamine formation by Glns in astrocytes (Cheng et al., 1972; Paulsen et al., 1987) and thereby may lower total GABA pool (Waagepetersen et al., 1999). Our studies also revealed that FA accelerated mIPSC deactivation both in NC and ANCC (Figures 6B/C). Recently, Christian and Huguenard (2013) reported a similar effect of fluorocitrate in thalamic reticular nucleus and attributed it to an allosteric modulation of α3 subunit-containing GABA_A_ receptors. However, in hippocampus, similar effects of astrocyte-released endozepines have not been described so far although diazepam binding inhibitor protein expression was reported in various brain structures including hippocampus (Yanase et al., 2002). Thus, a similar effect to that reported by Christian and Huguenard (2013) cannot be excluded in our model.

Taking altogether, we report that astrocytes strongly regulated GABAergic transmission by affecting mainly the frequency of mIPSCs. Our pharmacological data strongly suggest that this impact of astrocytes on GABAergic transmission involves regulation of key enzymes involved in cellular metabolism.

## Conflict of Interest Statement

The authors declare that the research was conducted in the absence of any commercial or financial relationships that could be construed as a potential conflict of interest.
